# Real-world evidence on efficacy and toxicity of targeted therapy in older melanoma patients treated in a tertiary-hospital setting

**DOI:** 10.1097/CMR.0000000000000997

**Published:** 2024-08-30

**Authors:** Ronen Stoff, Svetomir N. Markovic, Robert R. McWilliams, Lisa A. Kottschade, Heather N. Montane, Anastasios Dimou, Arkadiusz Z. Dudek, Winston Tan, Roxana S. Dronca, Mahesh Seetharam, Ruqin Chen, Matthew S. Block

**Affiliations:** a Department of Medical Oncology, Mayo Clinic Comprehensive Cancer Center, Rochester, Minnesota; b Department of Hematology and Oncology, Mayo Clinic Comprehensive Cancer Center, Jacksonville, Florida; c Department of Medical Oncology, Mayo Clinic Comprehensive Cancer Center, Scottsdale, Arizona, USA

**Keywords:** aged, melanoma, targeted therapy, toxicity

## Abstract

Melanoma is the deadliest form of skin cancer. The median age at diagnosis is 66. While most patients are treated with immunotherapy, the use of targeted therapy is a valid alternative for patients whose tumors harbor a BRAF or c-KIT driver mutation. These agents, while effective, come with a variety of side effects which limit their use, especially in older patients. We sought to assess the efficacy and toxicity of these agents in older melanoma patients. Melanoma patients over 65 treated with BRAF/MEK or c-KIT inhibitors were retrospectively identified, and their data were analyzed for treatment efficacy and toxicity. All data were compared using the Chi-square test for categorical comparisons and the Kruskal–Wallis method for median comparisons. One hundred and sixteen patients were identified. One hundred and six patients were treated with BRAF/MEK inhibitors. The assessed response rate (RR) was 83% and was comparable across different subgroups, including advanced line patients and those with a more aggressive disease. The median progression free survival (PFS) was 7.9 months, and the median overall survival (OS) was 15.7 months. Twenty-seven percent experienced grade 3–4 toxicity leading to a 24% treatment discontinuation rate. Another 10 patients were treated with the c-KIT inhibitor imatinib, for whom the assessed RR was 55%. The median PFS was 4.3 months, and the median OS was 22.6 months. Forty percent needed dose reductions, yet none had to stop treatment due to adverse effects. The use of targeted therapy in older patients is effective yet challenging due to toxicity. Deploying mitigation strategies can help maximizing their usefulness.

## Introduction

Malignant melanoma is the 5^th^ most common cancer diagnosed in the USA with about 100 000 new cases and 8000 deaths expected in 2024 [1]. The median age at diagnosis is 66, with the median age for melanoma specific mortality being 72 [2].

BRAF is the most common driver mutation in melanoma, seen in about 40–60% of all patients [3,4]. BRAF is a member of the MAPK pathway, and an activating mutation in BRAF constitutively activates the MAPK pathway via MEK and ERK signaling leading to cell proliferation and survival [5]. The most common BRAF mutation seen in melanoma is V600E which is present in about 80–90% of all BRAF mutated cases, with V600K accounting for another 15% and the less common V600D/G/R/M occurring in less than 5% of cases [6]. The presence of BRAF V600E mutation is the strongest predictor of response to BRAF and MEK inhibitors [7]. Other non-V600 mutations and BRAF fusions are found in about 4–14% of primary cutaneous melanoma cases [7], yet since they are part of the BRAF class II and III mutations they generally respond poorly to combined BRAF and MEK inhibition [7,8].

Currently approved BRAF-MEK combination therapies for unresectable/metastatic melanoma show high objective response rates (RRs) of about 65–70% and a disease control rate of about 90% [9–13]. The median progression free survival (PFS) is about 12 months with ≈20% of patients remaining progression free at the 5-year landmark. The two main shortcomings of these agents are the high frequency of acquired resistance and the variety of dose-limiting toxicities. The latter being a challenge for treating clinicians and patients alike, with constitutional side effects such as fever, fatigue, arthralgia as well as gastrointestinal, hepatic, and cardiovascular toxicities leading to dose interruptions, reductions, and discontinuations. In the pivotal combination therapy trials the treatment discontinuation rate due to adverse events was 12–18%.

Dabrafenib and trametinib is the only combination which has also been approved as adjuvant treatment for fully resected stage III melanoma patients [14]. Toxicity was the biggest limitation in this trial with a 26% discontinuation rate and 38% dose reduction rate due to adverse events [15].

Other driver oncogenes seen less frequently in melanoma are NRAS and c-KIT with activating mutations.

NRAS is the second most common driver oncogene and accounts for 15–20% of all cases, yet has only modest response to MEK inhibitors in the few clinical trials that have been published [16–18]. c-KIT mutations are most commonly seen in patients with mucosal or acral melanoma (15–20%) and in a about 2–3% of cutaneous melanomas that arise from chronic sun-damaged skin [19]. Existing scarce data show objective responses to KIT targeted therapy with imatinib in patients with Exon 11 or Exon 13 mutations [20–22] with no responses in patients with other loci of mutations or with c-KIT wildtype unselected patient populations [23,24]. Data also exist on responses to other c-KIT inhibitors including nilotinib [25,26], dasatinib [27], and sorafenib [28]. As with many other small molecule tyrosine kinase inhibitors, dose-limiting toxicities such as fatigue, nausea, diarrhea, muscle cramps, and prolonged QTc interval are frequent [29] and often require dose interruptions and modifications for many patients.

Given the fact that about 50% of all melanoma patients are over 65 at diagnosis, treatment with targeted therapy can be more challenging in this age group owing to significant comorbidities and frailty of many of these patients, limiting the clinical benefit from these agents. Many treating clinicians try to avoid using targeted therapy altogether or deploy preventive dose reduction strategies to minimize adverse effects and maximize treatment tolerability and efficacy. The effect of dose reductions and interruptions on treatment efficacy is mostly unknown, although previous trials comparing intermittent versus continuous dosing have shown that intermittent dosing seems to be associated with shorter PFS [30–32], thus implying that dose interruptions due to toxicity could potentially decrease treatment efficacy and duration of response.

Data regarding efficacy and toxicity of immunotherapy in older patients have become abundant in recent years [33–36], leading to widespread use of these agents in older and frail patients, which in the past were deemed unfit for systemic therapy. Yet, overall data regarding the efficacy and toxicity of targeted therapy in older patients are scarce [37] and mostly include subgroup analysis of the pivotal phase III trials, with only 24–29% of patients reported being older than 65 [9–13]. On top of that, the trials’ rigorous accrual process excludes many potential candidates and therefore they often do not reflect the complexity of real-world patient populations. In these trials there were conflicting data on the added value of combination therapy over single agent BRAF inhibitors in terms of PFS and overall survival (OS) in patients older than 65 years, mainly due to the small size of these cohorts [9–13]. It is worth noting that a pooled analysis of the dabrafenib and trametinib trials did show that an increase in the 10-year age increment was associated with better PFS and OS, yet the specific age cutoff was not mentioned [10].

In this retrospective analysis we sought to better characterize the outcomes of older melanoma patients with different targeted therapy agents aiming to provide insight on the best way to incorporate these agents in the treatment paradigm for elderly patients. The data generated in this publication have possible implications beyond melanoma patients following the tumor-type agnostic approval of dabrafenib and trametinib for solid tumors harboring BRAF V600E activating mutations in 2022 [38].

## Methods

Using the Epic Slice and Dice tool we have retrospectively identified the electronic medical records (EMR) of all advanced melanoma patients over 65 years of age that were treated with targeted therapy agents across the Mayo clinic enterprise (Rochester, Minnesota; Jacksonville, Florida; Scottsdale, Arizona, USA) between 2017 and 2023. All EMR of eligible patients were analyzed for demographic characteristics, disease related characteristics, antineoplastic treatment regimens utilized and treatment outcomes in terms of efficacy and toxicity. All demographic and disease related characteristics were extracted using the clinical data pull feature on research electronic data capture system (REDCap). Disease staging was done using the American Joint Committee on Cancer v.8 [39]. Staging was determined at initial diagnosis and reevaluated at the beginning of each line of systemic treatment. Treatment efficacy outcomes were done using the treating clinician’s assessment as was documented in the EMR, as well as imaging official interpretation documented in the EMR. The imaging methods analyzed were computerized tomography (CT) and PET/CT for systemic response assessment and CT and MRI for intracranial response assessment. When feasible, the efficacy was assessed using the RECIST criteria v1.1 [40]. Toxicity outcomes were collected based on documented notes by the treating clinician during office encounters and were based on patient reporting within those encounters. Accordingly, all toxicities documented were graded using the common terminology criteria for adverse events (CTCAE) v. 5.0 [41].

All data were recorded and managed using the REDCap system version 14.0 hosted at Mayo clinic [42,43]. REDCap is a secure, web-based application designed to support data capture for research studies, providing: (a) an intuitive interface for validated data entry; (b) audit trails for tracking data manipulation and export procedures; (c) automated export procedures for seamless data downloads to common statistical packages; and (d) procedures for importing data from external sources.

Categorical data comparison between subgroups was done using the Chi-square test. Medians of PFS and OS were compared using the Kruskal–Wallis method. For all analyses a *P*-value <0.05 was considered statistically significant.

All data were collected and analyzed under the approval of the Mayo Clinic Institutional Review Board (23-010095).

The manuscript was prepared according to the European society of medical oncology’s (ESMO) guidelines on reporting oncology real-world evidence [44].

## Results

**Fig. 1:**
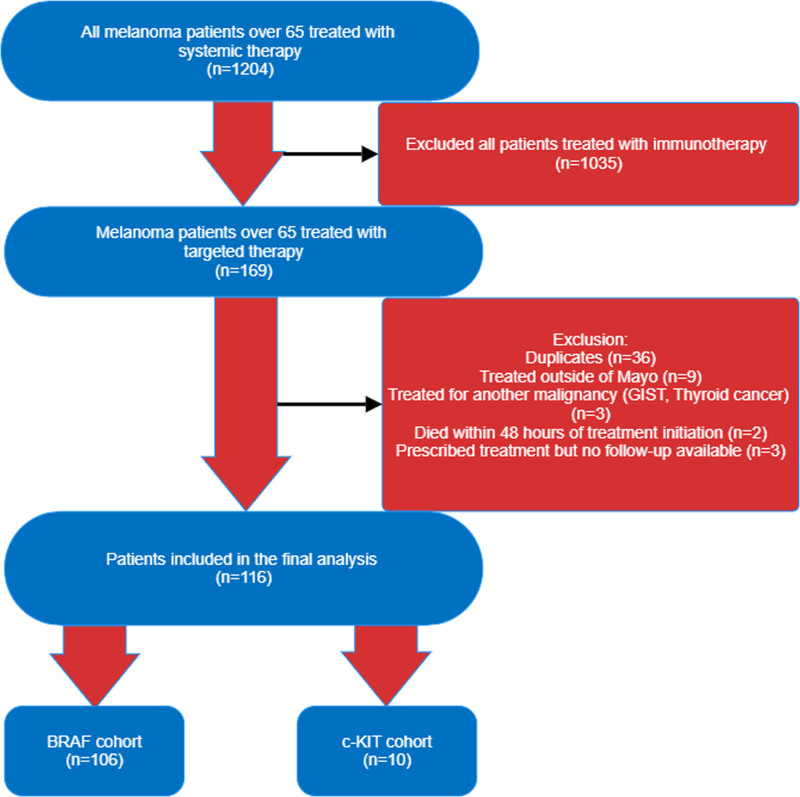
Flowchart of patient selection for the study.

A total of 116 patients over age 65 treated with targeted therapy were identified. These were further divided into two cohorts: 106 patients in the BRAF mutated cohort and 10 other patients in the c-KIT mutated cohort. A flow chart showing the patient selection process is shown in Fig. 1.

### BRAF cohort

One hundred and six BRAF mutated advanced melanoma patients were identified. BRAF mutation status was obtained using next generation sequencing (NGS) in all patients, and 99% had a documented BRAF V600E/K mutation (with the remaining one patient having an Exon 11 mutation). The median age was 71 (65–89) and 55% were males. The median follow-up was 28.9 months (1–124). Baseline patient characteristics are depicted in Table 1.

**Table 1:** Baseline patient characteristics – BRAF cohort

Characteristic	*N* (%)
Sex	
Male	59 (55%)
Female	47 (45%)
Treatment line	
1^st^	32 (30%)
2^nd^	58 (55%)
3^rd^	16 (15%)
Disease stage	
III or IV NED	22 (21%)
IV	84 (79%)
ECOG performance status	
0–1	67 (63%)
2–3	12 (11%)
Unknown	27 (26%)
LDH at treatment initiation	
WNL	76 (72%)
<X2 WNL	20 (19%)
>X2 WNL	10 (9%)
Agent used	
Dabrafenib + trametinib	61 (57%)
Encorafenib + binimetinib	37 (35%)
Vemurafenib + cobimetinib	2 (2%)
Single agent BRAF inhibitor	2 (2%)
Combination BRAF/MEK + anti PD-1/PDL-1	4 (4%)
CNS metastases at treatment initiation	
Yes	37 (35%)
No	69 (65%)
Melanoma subtype	
Cutaneous	75 (71%)
Acral	7 (7%)
Unknown	23 (22%)
Uveal	1 (<1%)
Comorbidities of interest	
Autoimmune disorders	20 (19%)
Type II diabetes	18 (17%)
Ischemic heart disease	12 (11%)
Atrial fibrillation	17 (16%)
Solid organ transplant recipient	3 (3%)

ECOG, Eastern Cooperative Oncology Group; LDH, lactate dehydrogenase; N/A, not applicable; NED, no evidence of disease; WNL, within normal limits.

Thirty-two (30%) patients were treated at first line, 58 (55%) at second line, and 16 (15%) at third line. Eighty-eight patients (83%) received treatment for unresectable/metastatic disease and were evaluable for their best radiological response. The assessed RR was 83%, of which 59% had a partial response (PR) and 24% had a complete response (CR). The disease control rate was 92%, 4% had a mixed response and only 4% had progressive disease (PD) as their best documented response. RR was 82, 83, and 87% for first, second, and third line, respectively. For responders, the median duration of response was 8 months (0.5–85) and the median time to achieve the best documented overall response was 3.1 months (0.7–27).

Of the evaluable patients, 28 (32%) had elevated serum lactate dehydrogenase (LDH) at treatment initiation, of which 86% responded to treatment (68% PR and 18% CR). The Eastern Cooperative Oncology Group performance status (ECOG PS) was 0–1 for 61% of the evaluable patients, 2–3 for 14% and unknown for the other 25%. RR was 89% for patients with ECOG PS 0–1 and 75% with those with ECOG PS of 2–3. Most patients (71%) had a cutaneous primary, another 22% had an unknown primary and 7% had acral primary. RR was 91% for cutaneous primary and 67% for acral primary. There was one uveal melanoma patient with an exon 11 BRAF mutation who did not respond to treatment.

Thirty-five percent of all patients had active central nervous system (CNS) metastases at treatment initiation, of which two patients were treated in the adjuvant setting following complete resection of the CNS disease, and therefore were not evaluable for response. For those who were evaluable, the systemic RR was 77%. Only 54% of all CNS patients were evaluable for intracranial response assessment, mostly due to administration of radiotherapy (stereotactic or whole brain) prior to or concomitantly with the initiation of systemic targeted therapy. The intracranial RR for the evaluable patients was 68%, and 39% for the entire CNS patient population. There were seven patients (20% of all CNS patients) who continued systemic treatment with targeted therapy beyond CNS progression while concomitantly undergoing salvage radiotherapy or neurosurgical metastasectomy.

The most common treatment regimen was dabrafenib and trametinib which was used in 57% of all patients (44% for metastatic/unresectable disease and 13% as adjuvant treatment), followed by the combination of encorafenib and binimetinib that was used in 35%. Only 2% were treated with vemurafenib + cobimetinib and another 2% were treated with a single agent BRAF inhibitor. RRs were similar for dabrafenib and trametinib and encorafenib and binimetinib with 83 and 85%, respectively. Four patients were treated with a combination of both targeted therapy and an anti PD-1 agent with all of them achieving a response (3 PR and 1 CR).

RR comparisons according to subgroups are summarized in Table 2.

**Table 2:** Response rate according to subgroup analysis BRAF cohort

Subgroup (*n*) – evaluable only	Response rate^a^ *n* (%)	*P*-value
Male (51)	42 (82%)	
Female (37)	31 (84%)	*P* = 0.86
Normal LDH (60)	49 (82%)	
Elevated LDH (28)	24 (86%)	*P* = 0.63
ECOG PS 0–1 (54)	47 (87%)	
ECOG PS 2–3 (12)	9 (75%)	*P* = 0.29
Dabrafenib + trametinib (47)	38 (81%)	
Encorafenib + binimetinib (33)	28 (85%)	*P* = 0.64
1^st^ line (22)	18 (82%)	Reference
2^nd^ line (47)	39 (83%)	*P* = 0.9
3^rd^ line (16)	14 (87%)	*P* = 0.63
CNS disease (35)	27 (77%)	
No CNS disease (47)	40 (85%)	*P* = 0.35
Age over 75 (11)	7 (64%)	
Age 65–75 (77)	66 (86%)	*P* = 0.068

CNS, central nervous system; ECOG PS, Eastern Cooperative Oncology Group performance status; LDH, lactate dehydrogenase.

a Systemic response rate only.

At data cutoff, 13% of patients were on treatment with the other 87% stopping treatment. The most common reason for treatment discontinuation was disease progression or death for 53% of all patients. Twenty-one percent of patients discontinued treatment due to adverse events and the other 13% discontinued treatment at a preplanned time point – either at the end of 1 year of adjuvant treatment or at the discretion of the treating clinician.

The median PFS was 7.9 months, with a PFS of 6, 8.3, and 13.4 months for first, second, and third line, respectively. There was not statistically significant difference in PFS among different lines of treatment. The median PFS was 5.7 months for patients with active CNS disease.

At data cutoff 53 patients (50%) have died, with melanoma being the cause of death for 85% of them. The median OS from time of treatment initiation was 15.7 months (12.5, 14.5, 31.9 for first, second, and third line, respectively). There was not a statistically significant difference in OS among different lines of treatment. The median OS for patients with CNS disease was 9.8 months.

Thirteen patients (12%) were over the age of 75 at treatment initiation, with a median age of 79 (75–88). All patients were female. In this age group the combination of encorafenib and binimetinib was used in 61% of patients with the other 39% receiving treatment with dabrafenib and trametinib. Eleven patients were evaluable for response with an assessed RR of 64%. Only one patient had CNS disease which did not respond to treatment. Of the 11 patients, 1 (9%) was still on treatment at data cutoff, with 7 (64%) stopping treatment due to disease progression or death, 2 (18%) due to adverse events, and the other 3 (27%) at the discretion of the treating physician. The median PFS was 9.2 months (4–18), and the median OS was 11.7 months (1.8–41).

Eighty-eight percent of patients experienced adverse events of any grade, with 27% having grade 3–4 toxicity. There were no grade 5 toxicities reported. Thirty-six percent of patients experienced one adverse event, while 32% experienced two and 33% experienced three or more. Twenty-one percent of patients stopped treatment due to toxicity, 28% needed a dose reduction, and 20% changed to a different set of BRAF/MEK inhibitors. Of the patients who changed to a different combination of agents – 76% experienced adverse events from the new regimen, leading to dose reduction in 69% of them and permanent treatment discontinuation in another 19% (leading to a total of 24% discontinuation rate).

While most adverse events were treated with palliative care and dose interruptions or reductions, about 10% of patients needed systemic corticosteroids to treat adverse events, with fever (27%) and elevated liver function tests (27%) being the most common causes.

The most common toxicities seen were fever (30%), fatigue (30%), skin rash (22%), elevated liver function tests (20%), arthralgia/myalgia (15%), nausea (14%), and diarrhea (11%). Seven percent had ejection fraction decrease with one grade 3 leading to permanent treatment discontinuation.

### c-KIT cohort

Ten patients with NGS proven c-KIT mutated melanoma were identified. All patients were treated with imatinib. The median age at treatment initiation was 75 years (71–100). Three patients had cutaneous primary, 3 had vulvar/vaginal primary, 3 had acral primary, and 1 had anal canal primary. All patients had normal LDH at treatment initiation and ECOG performance status was 0–2 (median 0). Baseline patient characteristics are depicted in Table 3.

**Table 3:** Baseline patient characteristics – c-KIT cohort

Characteristic	*N* (%)
Sex:	
Male	5 (50%)
Female	5 (50%)
Treatment line:	
Adjuvant	1 (10%)
2^nd^	6 (60%)
3^rd^	3 (30%)
ECOG performance status	
0–1	8 (80%)
2	1 (10%)
Unknown	1 (10%)
Primary site	
Acral	3 (30%)
Cutaneous	3 (30%)
Vulvar/vaginal	3 (30%)
Anal canal	1 (10%)
KIT mutation location:	
Exon 11	6 (60%)
Exon 13	1 (10%)
Exon 14	1 (10%)
Exon 17	2 (20%)

ECOG PS, Eastern Cooperative Oncology Group performance status.

One patient received treatment with imatinib as off-label adjuvant treatment for stage IIIB disease. This patient was treated for 18 months with no evidence of recurrence. The patient discontinued treatment after 18 months at the treating physician’s discretion and continued follow-up only to have a recurrent metastatic disease 6 months after treatment discontinuation, at which point the patient elected for best supportive care. The other nine patients received imatinib for stage IV disease with an overall assessed RR of 55% (44% PR, 11% CR). Median duration of response was 4 months (3–39). Responses were seen in different histological subtypes including: acral melanoma (67%), vulvar/vaginal melanoma (67%), and cutaneous melanoma (33%). All nine metastatic patients were treated with immunotherapy at first or second line before proceeding to treatment with imatinib. Six patients were treated as 2^nd^ line with an RR of 67% and three were treated as 3^rd^ line with an RR of 33%. There was only one patient with CNS disease who did not respond to treatment either systemically or intracranially. Six patients had Exon 11 mutations (V560D/G/E, N564S, and L576P) of which four responded (67%). Two had Exon 17 mutations (N822C/K) of which one responded (50%) and the remaining two had an Exon 14 (F681I) and Exon 13 (K642E) mutation that did not respond to treatment at all.

At data cutoff two patients were on treatment and seven had stopped treatment due to disease progression or death. Median PFS was 4.3 months (1.8-not reached), median OS from treatment initiation was 22.6 months (4–64).

Toxicity was moderate with 90% of patients experiencing any grade adverse event, with the median grade being 1 (1–3). Fifty percent of patients experienced two adverse events, 20% had three adverse events, and the other 20% had four adverse events. Only one patient experienced toxicity requiring dose interruption with two grade 3 toxicities – pneumonitis and decreased neutrophil count, leading to temporary treatment interruption, dose reduction, and a course of corticosteroids. The rest of the patients experienced only grade 1–2 toxicity, yet in total four patients (40%) needed dose reductions. there were no treatment discontinuations due to toxicity and there were no treatment related deaths.

## Discussion

Age remains the biggest risk factor for most solid cancers, with about a half of all melanoma patients being diagnosed at 65 years of age or older [2]. Additionally, the life expectancy is growing which means the absolute number of older patients being diagnosed with melanoma is increasing [1]. Until a decade ago the treatment options for melanoma patients were very limited and consisted mainly of highly toxic and poorly effective regimens based on chemotherapy [45–47] alone or in combination with immunotherapy with high doses of interleukin-2 (IL-2) [48,49] or interferon alpha (IFNa) [50]. As many older patients are more prone to be frail and/or suffer from multiple comorbidities, they were often considered poor candidates for such aggressive treatment regimens, and therefore were mainly offered supportive care.

The introduction of novel immune check point inhibitors (ICI) has revolutionized the treatment paradigm in melanoma, yet at the cost of various immune mediated toxicities [51,52]. Nevertheless, clinicians and patients alike seem eager to use these agents even in older and frail patients with available data showing that, overall, these agents are both efficacious and safe for such patients [33–36]. While ICI is the primary treatment method used for melanoma, not all patients benefit from these agents. The main limitations remain treatment resistance (primary or acquired) and toxicity, especially for solid organ transplant recipients and patients with preexisting autoimmune disorders [53–55].

The alternative to ICI is targeted therapy aimed at driver mutations, with BRAF being the most prevalent mutation in cutaneous melanoma and c-KIT being the most prevalent mutation in mucosal melanoma.

Anti BRAF + MEK treatment combinations have become widely used simultaneously to the introduction of ICI. Their biggest value is the rapid response seen in a high proportion of patients, which is especially useful in cases of progressive multifocal metastatic presentation. With that being said, the two biggest limitations remain acquired resistance and various toxic side effects. The issue with the limited duration of response remains an unmet need and leads to preference of ICI as the first-line modality when feasible, as seen in trials assessing optimal treatment sequencing [56] and as reflected in the national comprehensive cancer network (NCCN) guidelines [57]. In addition, the various dose-limiting toxicities are challenging to manage, and require dose interruptions and reductions in many cases. This is especially true in older and more frail patients, who might take longer time to recover from such toxicities and therefore might need longer treatment interruptions, which can hamper the efficacy and duration of response.

The data collected in our real-world evidence cohort of BRAF mutated patients reflect a diverse population of melanoma patients older than 65 years, which are usually not fully represented in pharma based clinical trials. Our data suggest that toxicity can be challenging in older patients, with 24% treatment discontinuation due to toxicity, which is higher than reported in the pivotal clinical trials of about 12–18% in the metastatic setting and similar to the 26% reported in the adjuvant setting. No specific data on the prevalence and characteristics of toxicity in older patients were published in any of the pivotal trials [9–13].

A significant portion of patients also required dose reductions and switching to other BRAF + MEK combinations, yet the possibility of both interventions increases the ability to manage side effects without foregoing treatment in most patients. Efficacy is higher than reported in trials, though our assessment was not done according to RECIST v1.1 and therefore cannot be directly compared to the rates shown in the pivotal trials. It is worth noting that most patients were treated with targeted therapy as a 2^nd^ or 3^rd^ line, and for those patients the PFS and OS seems to be numerically longer than for those treated in the first-line setting. This was not statistically significant, most likely due to the small sample size. These differences can be explained by the fact that those patients who receive first-line treatment with targeted therapy are usually the ones who present with a rapidly aggressive disease, often with a significant disease burden and CNS involvement. This is also reflected in the fact the median PFS and OS for first-line patients in our study were shorter than the ones reported in the pivotal trials [9–13]. The responses observed in our cohort show that targeted therapy is effective in all various high risk disease factors including elevated LDH, poor ECOG PS, and CNS involvement. The issue with duration of response is still an open one, even though a small portion of the patients have prolonged and durable responses.

In our c-KIT mutated cohort we bring the first series of older melanoma patients treated with imatinib, which shows encouraging results in terms of RR, especially given that all patients received treatment as a 2^nd^ or 3^rd^ line following ICI. Responses seem to rely heavily on the location of the mutation within the c-KIT gene, which can help for better patient selection in the future. The duration of response is short, as previously described and remains an unmet need for this patient population. Hopefully, future data collected on the use of other KIT inhibitors will show promising results in terms of response and durability. The most encouraging outcome for this cohort was the milder toxicity profile which allowed all patients to receive optimal treatment duration, which should encourage the routine testing for c-KIT mutation in BRAF wild type patients and the incorporation of imatinib and other KIT inhibitors in the treatment paradigm for selected KIT mutated patients, with an emphasis on the location of the mutation as a predictive factor.

The results of this study should be interpreted with caution as this is a retrospective study reflecting a selected population of patients treated in a high volume tertiary medical center. The limitations of this study are mainly because all data were extracted from EMRs, in which the medical notes are not uniform in the style and depth of coverage. This is the case for toxicity assessment which relies mainly on patient reporting during medical encounters, which might be influenced by a reporting or recall bias. Since in the real-world setting most care takers do not routinely grade toxicity, the grading done by our team can also be inaccurate or incomplete. Another limitation is with response assessment, which is related to the fact the RECIST criteria are not routinely used in the real-world setting by both clinicians and radiologists. This is further complicated by the fact that different imaging modalities were assessed, which can lead to inaccurate assessment of response patterns, especially when comparing PET/CT and CT. Data suggest that metabolic response assessment by PET/CT is faster than conventional anatomical CT response [58], yet at times metabolic changes can also reflect immune responses, even in patients treated with targeted therapy [59].

### Conclusion

Targeted therapy is a valid treatment option for older BRAF mutated melanoma patients, preferably after exposure to ICI when medically feasible. The biggest challenge remains toxicity, yet a variety of tactics deployed such as dose interruptions, reductions, and agent switching can help mitigate this obstacle, thus allowing patients a proper exposure to these agents. These data can also be reproduced for other older BRAF mutated cancer patients as the use of BRAF + MEK inhibitors becomes increasingly popular.

For c-KIT mutated patients, treatment with imatinib and other KIT inhibitors seems appropriate post ICI exposure, while harnessing the predictive value of mutation location within the c-KIT gene.

Further studies and real-world data are needed to better allow for patient selection for targeted therapy at different stages and lines.

## Acknowledgements

The statistical planning and analysis were performed with the gracious assistance of the Mayo Clinic center for clinical and translational science (CCATS) biostatistics, epidemiology, and research design resource (BERD).

This publication was supported by Grant Number UL1TR002377 from the National Center for Advancing Translational Sciences (NCATS). Its contents are solely the responsibility of the authors and do not necessarily represent the official views of the NIH.

### Conflicts of interest

R.S. received speaker fees from Bristol-Myers Squibb, Merck Sharp Dohme, Novartis, Medison Pharma and is a consultant for Pangea Biomed; S.N.M. reports institutional research support from Bristol-Myers Squibb and Sorrento Therapeutics; M.S.B. reports institutional research support from Alkermes, Bristol-Myers Squibb, Genentech, Marker Therapeutics, Merck, nFerence, Pharmacyclics, Regeneron, Sorrento, TILT Biotherapeutics, Transgene, and Viewpoint Molecular Therapeutics and is a consultant/advisory board member (unpaid) for Marker Therapeutics, Sorrento Therapeutics, TILT Biotherapeutics, and Viewpoint Molecular Targeting. For the remaining authors, there are no conflicts of interest.
